# Health outcomes for Australian Aboriginal and Torres Strait Islander children born preterm, low birthweight or small for gestational age: A nationwide cohort study

**DOI:** 10.1371/journal.pone.0212130

**Published:** 2019-02-20

**Authors:** Elizabeth M. Westrupp, Fabrizio D'Esposito, Jane Freemantle, Fiona K. Mensah, Jan M. Nicholson

**Affiliations:** 1 Judith Lumley Centre, La Trobe University, Melbourne, Victoria, Australia; 2 School of Psychology, Deakin University, Burwood, Melbourne, Victoria, Australia; 3 University of Melbourne, Victoria, Australia; 4 Murdoch Children’s Research Institute, Melbourne, Victoria, Australia; 5 Royal Children’s Hospital, Melbourne, Victoria, Australia; Centre Hospitalier Universitaire Vaudois, FRANCE

## Abstract

**Objective:**

To examine health outcomes in Australian Aboriginal and Torres Strait Islander children experiencing perinatal risk and identify protective factors in the antenatal period.

**Methods:**

Baby/Child cohorts of the Longitudinal Study of Indigenous Children, born 2001–2008, across four annual surveys (aged 0–8 years, N = 1483). Children with ‘mild’ and ‘moderate-to-high’ perinatal risk were compared to children born normal weight at term for maternal-rated global health and disability, and body-mass-index measured by the interviewer.

**Results:**

Almost one third of children had experienced mild (22%) or moderate-to-high perinatal risk (8%). Perinatal risk was associated with lower body-mass-index z-scores (regression coefficients adjusted for pregnancy and environment factors: mild = -0.21, 95% CI = -0.34, -0.07; moderate-to-high = -0.42, 95% CI = -0.63, -0.21). Moderate-to-high perinatal risk was associated with poorer global health, with associations becoming less evident in models adjusted for pregnancy and environment factors; but not evident for disability. A range of protective factors, including cultural-based resilience and smoking cessation, were associated with lower risk of adverse outcomes.

**Conclusions:**

Perinatal risks are associated with Australian Aboriginal and Torres Strait children experiencing adverse health particularly lower body weight. Cultural-based resilience and smoking cessation may be two modifiable pathways to ameliorating health problems associated with perinatal risk.

## Introduction

Data from two Aboriginal cohorts suggest a long-term health burden associated with perinatal risk in Aboriginal and Torres Strait Islander children and adults born prior to 1990.[1–4] It is important to investigate these associations for more recent cohorts of Indigenous Australian children, to determine whether these health disparities persist in contemporary cohorts twenty years after these patterns were first identified and calls for change widely discussed.[5, 6] The social and economic context of Australian Indigenous families is fundamental to understanding child health outcomes.[7, 8] Australian Aboriginal and Torres Strait Islander people have experienced generational disadvantage and discrimination for over 200 years, and consequently have suffered significant social and health inequities.[7, 8] In line with the National Aboriginal and Torres Strait Islander Health Plan in Australia, we focus on the social, cultural and economic protective factors that promote positive health and development in Aboriginal and Torres Strait Island communities for children born with perinatal risk since 2000.[9, 10] We test whether a range of child, birth, pregnancy, social and maternal factors offer protection against adverse health outcomes in the context of perinatal risk.

Findings from the Aboriginal Birth Cohort Study showed that children born intrauterine growth restricted (small for gestational age) in 1987–1990 were 2cm shorter and 4kg lighter than peers at age one;[3] and birthweight was negatively associated with child blood pressure, but not with other measures of chronic disease at 11 years.[4] Evidence from a study of adults born before 1985 in an Aboriginal community in the Northern Territory showed an association between low birthweight and higher rates of mortality, high blood pressure and diabetes.[1, 2] Studies of Indigenous populations in Canada, New Zealand and the USA also suggest a link between birth weight and type 2 diabetes, impaired kidney function and high blood pressure.[11]

The current study uses data from Footprints in Time, the Longitudinal Study of Indigenous Children (LSIC). LSIC is funded and conducted by the Australian government and in the current paper is accessed for the purpose of secondary data analysis. We investigate health outcomes for Aboriginal and Torres Strait Islander children born in 2001–2008 with ‘mild’ and ‘moderate-to-high risk’ in regards to preterm birth, birthweight and size for gestational age. Comparisons are made with children in the same cohort born full-term with healthy birthweight. We investigate associations between perinatal risk and three childhood physical health outcomes. Consistent with findings from previous cohort studies, we expect that compared to children born at full-term, children with mild or moderate-to-high perinatal risk will be at increased risks of disability, physical health problems and a lower body mass index in early childhood. We expect these associations to be most evident for children with moderate-to-high compared to mild perinatal risk.

## Methods

### Study design

Footprints in Time: The Longitudinal Study of Indigenous Children is a prospective cohort study which aims to improve the understanding of the strengths and challenges experienced by Aboriginal and Torres Strait Islander children and families. A non-random, purposive sampling design was employed, with sample sites selected to be broadly representative of the socio-economic and community environments where Aboriginal and Torres Strait Islander children live.[12] Eligible families were approached and informed consent obtained.

This paper uses four time-points of data from the Baby (N = 960) and the Child cohorts (N = 727), aged 3–60 months (Baby cohort) and 24–68 months (Child cohort) at recruitment in 2008, and followed annually in 2009, 2010, 2011, and 2012; comprising 2% of the estimated Indigenous child population aged 0–5 years in Australia at the time of recruitment.[13] On average the intervals between adjacent assessments were 9.3, 12.2 and 11.2 months for each of the time-points respectively.[14] A change in child health measures collected at wave 5 prohibited the use of further time-points. Data were collected via face-to-face interviews with the primary carer of the study child (~93% mothers) and via direct child assessment undertaken by interviewers during home visits. Ethical approval was obtained from the Australian Government Department of Health and Ageing Departmental Ethics Committee. LSIC data are openly accessible but prospective users must complete a deed of licence and sign a LSIC Data Integrity Statement in order to access data. Information is available at https://www.dss.gov.au/about-the-department/publications-articles/research-publications/longitudinal-data-initiatives/footprints-in-time-the-longitudinal-study-of-indigenous-children-lsic#3.

### Participants

**Fig 1:**
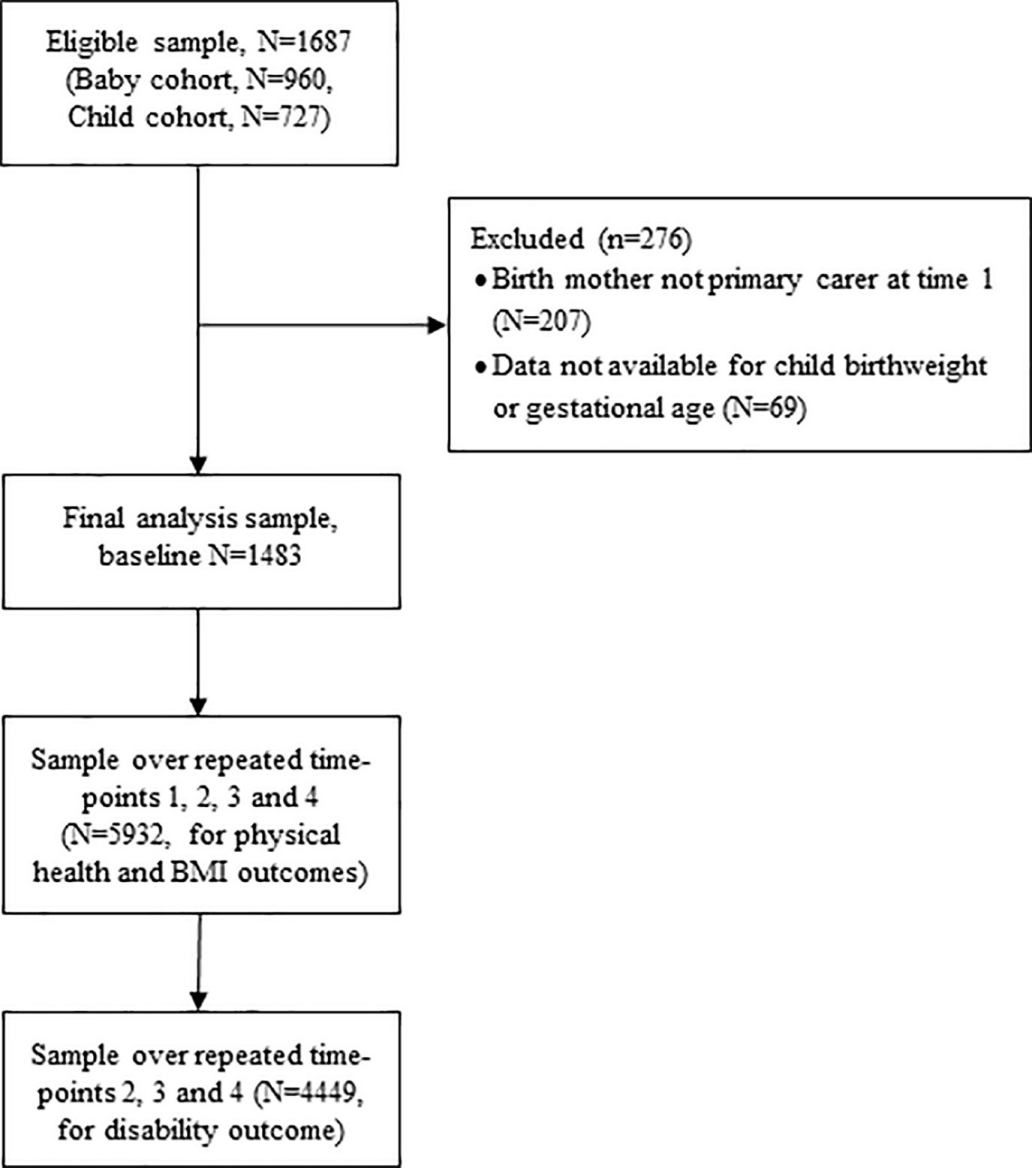
Flow diagram showing the selection of participants into the final analysis sample.

All analyses were conducted using combined data from the Baby and Child cohorts. A flow diagram showing the final analysis sample is presented in Fig 1. Inclusion criteria for this paper required that the birth mother was the primary carer at time 1 (N = 207 excluded), and that data were available for either child birthweight or gestational age (N = 69 excluded); resulting in a final sample of 1483 mothers and their children. Children were classified into one of three groups. The ‘moderate-to-high perinatal risk’ group if they were born very preterm (<32 weeks), extremely small for gestational age (SGA, ≤1st percentile for weight by gestational age/gender, calculated using Australian birthweight percentiles 1998–2007),[15] and/or very low birthweight (<1500g). The ‘mild perinatal risk’ group were born at 32–36 weeks’, were SGA (2^nd^-9^th^ percentile), and/or had birthweight 1500-2499g. Classification was based on the category that placed the child at greatest risk, regardless of whether this was based on one or all three measures of perinatal risk. The full-term group were those children born term (≥37 week’s gestation), normal birthweight (≥2,500g), and were not small for gestational age (≥10^th^ percentile).

### Outcome measures

#### Physical health

The overall health of the study child was measured at four annual interviews times using one item from the SF-12[16] which assesses perceived global function and wellbeing as follows “Now I‘d like to ask some questions about your child’s health. In general, would you say your child’s health is excellent, very good, good, fair or poor?” Children were categorised on a binary variable as having excellent or very good health compared to good, fair or poor health. Interviewers followed a script to respond to queries or requests for clarification from the parents. For this item, the interviewer could reword the question slightly to: “Is <Study Child>‘s health always good, or are they sick a lot of times?”

#### Disability

Mothers reported the presence of intellectual, learning, physical, neurological, developmental, speech, psychiatric, acquired brain injury, and/or other disabilities at three time-points (time 2 to 4) only. Children were categorised as having a disability or not. There was no script available for interviewers to provide further clarification on this question but mothers could view the condition types displayed on a laptop computer.

#### Body mass index (BMI)

Interviewers measured children’s weight and height in the home at four annual interviews using standardized equipment and procedures. Children’s BMI (kg/m^2^) was then converted into age- and gender-specific z-scores based on the World Health Organisation Multicentre Growth Reference Study.[17]

### Covariates

Where applicable, covariates were coded to be strengths-based in order to test each variable as a possible protective factor. At the baseline assessment, mothers reported on the study child’s age and gender, singleton versus multiple birth, their own age, and their smoking or alcohol use during pregnancy (recoded to non-smoking and no alcohol use during pregnancy). At every time-point, mothers reported on their employment status, which was recoded to “in paid employment” (Yes; No). At time 2, mother’s reported on their high school education level, i.e., whether they had completed year 12 or equivalent (Yes; No). Level of relative (geographic) isolation was classified using the Accessibility/ Remoteness Index of Australia,[18] and recoded to represent accessible geographical locations (i.e., no or low remoteness) compared to remote locations (i.e., moderate or high/extreme remoteness).

Social and emotional distress was measured using the Social-Emotional Wellbeing subscale from the Strong Souls Questionnaire,[19] a culturally-sensitive tool developed in consultation with Indigenous people, and collected at each time-point. Seven items asked how often mothers felt symptoms of anxiety or depression in the last 4 weeks on a four-point likert scale (‘not much’ to ‘lots’). The scale was reversed so that higher scores indicated higher wellbeing.

Cultural resilience was assessed using the Resilience subscale of the Strong Souls Questionnaire, measuring culturally-related resilience and support.[19] 12 items from the Strong Souls Questionnaire were measured at time 1 and time 4, but were not available at times 2 and 3. Thus, data from time 1 was used to adjust for time 1 and 2 outcomes; and data from time 4 was used to adjust for time 3 and 4 outcomes. Higher scores indicate resilience.

Stressful life events were assessed using eleven items at each time asking about stressful events occurring in the last year, including pregnancy, new job, loss of a job, return to study, worry about money, housing problems, serious injury, illness or assault, death of family member or close friend, alcohol or drug problem, being mugged or assaulted, arrested or involvement with police, ‘humbugged’ (i.e., harassed for money). The 11 items from this scale were summed to form a total score where higher scores equated to more stressful life events.

### Statistical analyses

Analyses were conducted using Stata 13.1 with combined data from the Baby and Child cohorts. Differences between full-term and defined perinatal risk groups on sample characteristics were investigated using logistic and linear regression. Health outcomes were compared for full-term and perinatal risk groups, and unadjusted group differences reported (p for the trend across severity of perinatal risk) from logistic regression (disability and global health ratings) and linear regression (BMI) analyses. All regression analyses included repeated health outcomes measures for each child across the waves in which they had participated. The correlation between repeated outcome measures was accounted for using information sandwich (‘robust’) estimates of standard error. Interaction tests were conducted to evaluate whether there was evidence that associations varied by child age.

Two sets of multivariable regression models were performed to investigate the association between mild or moderate perinatal risk and child health outcomes while accounting for child and other protective (pregnancy, social and maternal) factors. The first model accounted for child age, gender, and singleton births only (Model 1). The second fully adjusted model accounted for child age, gender, singleton births; mother report of non-smoking and no alcohol use during pregnancy, higher social-emotional functioning, cultural-based resilience, and lower life event stress; and maternal age, education, not geographically isolated, Indigenous status, and maternal employment (Model 2).

### Missing data

The rate of missing data ranged from 0% to 35%, where the majority of model variables had <20% missing. Mothers with missing data were more likely to have older children, to identify themselves as Aboriginal or Torres Strait Islander, and to have used alcohol during pregnancy. Multivariate multiple imputation using chained equations was performed. All variables in the analytic models were included in the multiple imputation model to create 50 imputed datasets. Sample sizes varied for outcomes given that data for disability was not available at time-point 1. Final samples included N = 5932 assessments for global physical heath and BMI, and N = 4449 assessments for disability. The pattern of results did not differ between imputed data and complete case analysis, therefore imputed data are reported.

## Results

### Sample characteristics

Characteristics of the final sample by full-term and the two perinatal risk groups are shown in Table 1. One in five children (21.9%) were in the mild perinatal risk category, and 7.6% were in the moderate-to-high perinatal risk category. There were higher rates of singleton birth in the full-term children compared to perinatal risk groups, but distributions for child gender were similar. Rates of maternal non-smoking and no alcohol use during pregnancy were higher in the full-term group, and lowest in the moderate-to-high perinatal risk group. More mothers in the perinatal risk groups identified as having an Aboriginal and Torres Strait Islander background. There were no evident differences between full-term and perinatal risk groups on child age, maternal age, education, geographical location or employment status; or on maternal socio-emotional wellbeing or cultural-based resilience. However, mothers in the full-term group reported fewer stressful life events compared to mothers in the perinatal risk groups.

**Table 1:** Sample characteristics for full-term and perinatal risk (PR) groups.

	Full-term	Mild PR	Moderate-to-high PR	P for the trend
	*(71*.*1%)*	*(21*.*5%)*	*(7*.*4%)*	
*Baseline characteristics (time 1)*	*%*	*%*	*%*	
Singleton birth (versus multiple)	99.0	96.3	95.7	**0.002**
Child female	48.8	48.7	52.1	0.65
Non-smoking pregnancy	55.7	39.8	36.2	**<0.001**
No alcohol used in pregnancy	78.9	72.5	68.1	**0.003**
Maternal education Year 12 or above ^a^	46.6	40.7	35.0	**0.02**
Mother Aboriginal or Torres Strait Islander	80.3	87.8	87.2	**0.005**
Accessible geographical location	81.4	75.6	74.5	**0.02**
*Characteristics averaged over the assessments (times 1–4)*				
Child age in months (mean, se)	45.78 (0.36)	48.57 (0.71)	47.22 (1.26)	0.35
Mother age in years (mean, se)	30.38 (0.11)	30.51 (0.22)	30.30 (0.38)	0.89
Social-emotional wellbeing (mean, se)	17.27 (0.06)	16.96 (0.14)	16.69 (0.25)	**0.04**
Cultural-based resilience (mean, se)	40.47 (0.10)	40.53 (0.20)	39.62 (0.37)	0.19
Two or fewer stressful life events	40.2	36.1	33.7	**0.04**
Mother in paid employment	64.5	66.8	70.7	0.15

*Notes*: Data in table are from data including multiple imputation. P-values are from logistic or linear regression comparing sample outcomes by full-term and perinatal risk groups (p for the trend across full-term, mild, and moderate-to-high perinatal risk groups). Bold-face represents statistically significant results (p<0.05).

^a^Data for maternal education were collected at time 2.

### Perinatal risk and child health outcomes

Across the full sample, child health was rated by mothers as very good or excellent in 75.2% of the assessments, while in 2.2% of assessments, mothers reported that their child had a disability. BMI z-scores for this sample of children (mean = 0.57; SE = 0.02) were higher than World Health Organisation 2006 norms (i.e., compared to mean = 0.0)[20]. Table 2 presents descriptive data for child outcomes across the full-term and perinatal risk groups. A greater proportion of children in the mild and moderate-to-high perinatal risk groups were rated by their mothers as having fair, poor or good health compared to children born full-term (with a healthy birthweight). There were no differences between the full-term and mild perinatal risk groups on rates of disability. A strong gradient was evident for BMI z-scores, where both perinatal risk groups had lower mean scores compared to the full-term group, and children in the moderate-to-high group had the lowest scores overall. Underweight and overweight BMI categories based on WHO guidelines also demonstrated a gradient whereby children with mild and moderate-to-high perinatal risk were more likely to be underweight and less likely to be overweight.[17] These differences were most evident in older children (aged 5 or above).

**Table 2:** Child health outcomes by perinatal risk (PR) groups.

	Full-term	Mild PR	Moderate-to-high PR	P for the trend
Fair, poor or good global physical health	23.6%	26.2%	29.5%	**0.046**
Disability	2.2%	2.3%	2.8%	0.72
Body mass index z-score, mean (se)	0.66 (0.03)	0.41 (0.05)	0.18 (0.09)	**<0.001**
Body mass index WHO categories				
Underweight				
*Below age 5*	2.39	3.53	4.25	
*Age 5 and above*	2.16	3.74	9.42	
Risk of overweight				
*Below age 5*	62.03	59.80	59.63	
*Age 5 and above*	51.67	52.01	45.23	

*Notes*: Data in table are from data including multiple imputation and are averaged across the four assessment time-points. P-values are from logistic (global health and disability) or linear (BMI) regression analyses comparing sample outcomes by full-term and perinatal risk groups (p for the trend across full-term, mild, and moderate-to-high perinatal risk groups). Bold-face represents statistically significant results (p<0.05).

Table 3 presents odds ratios and regression coefficients with 95% confidence intervals for each of the health outcomes over the two sets of adjusted models. There was no evidence that associations varied by child age using a test of interaction, so estimates reflect children across all ages and repeated assessments. Compared to children born full-term with healthy birthweight, mild perinatal risk was not associated with poorer global health or disability outcomes. Moderate perinatal risk was associated with higher odds of good, fair or poor global health compared to very good or excellent global health (but not disability) in both adjusted models, although large variability in the data meant that this association was not statistically significant. The association between perinatal risk and lower child BMI remained stable across all adjusted models. In the final fully adjusted models (Model 2), both mild and moderate-to-high perinatal risk was associated with lower child BMI. The comparison between children in the full-term and moderate-to-high perinatal risk group equated to a half standard deviation average difference in BMI.

**Table 3:** Mild and moderate-to-high perinatal risk (compared to full-term) as predictors of child physical health outcomes across five sets of adjusted models.

		*Child Physical Health Outcomes*											
		Poor global physical health (N = 5932 assessments over four years)				Disability (N = 4449 assessments over three years)				Higher BMI (N = 5932 assessments over four years)			
*Adjusted Models*		*OR [95% CI]*			*p*	*OR [95% CI]*			*p*	*Coef [95% CI]*			*p*
1.	*Perinatal risk*												
	Full-term (reference)												
	Mild	1.15	[0.93,	1.41]	0.20	0.96	[0.51,	1.79]	0.90	**-0.24**	**[-0.38,**	**-0.10]**	**0.001**
	Moderate-to-high	1.37	[0.98,	1.92]	0.06	1.19	[0.52,	2.72]	0.68	**-0.47**	**[-0.69,**	**-0.26]**	**<0.001**
	*Child factors*												
	Child age	1.00	[0.99,	1.00]	0.18	**0.98**	**[0.96,**	**0.99]**	**0.01**	**-0.02**	**[-0.02,**	**-0.01]**	**<0.001**
	Child female	0.91	[0.77,	1.07]	0.23	**0.57**	**[0.34,**	**0.95]**	**0.03**	-0.09	[-0.20,	0.02]	0.12
	Singleton birth	1.40	[0.66,	3.00]	0.38	0.35	[0.09,	1.31]	0.12	-0.30	[-0.78,	0.17]	0.21
2.	*Perinatal risk*												
	Full-term (reference)												
	Mild	1.09	[0.88,	1.34]	0.44	1.02	[0.54,	1.91]	0.96	**-0.21**	**[-0.34,**	**-0.07]**	**0.004**
	Moderate-to-high	1.27	[0.91,	1.78]	0.16	1.20	[0.51,	2.80]	0.68	**-0.42**	**[-0.63,**	**-0.21]**	**<0.001**
	*Child factors*												
	Child age	1.18	[0.96,	1.46]	0.11	1.21	[0.67,	2.16]	0.53	0.10	[-0.03,	0.23]	0.14
	Child female	1.00	[0.84,	1.19]	0.99	1.14	[0.68,	1.92]	0.61	**-0.17**	**[-0.28,**	**-0.05]**	**0.005**
	Singleton birth	0.99	[0.97,	1.02]	0.56	1.05	[0.98,	1.11]	0.17	0.005	[-0.01,	0.02]	0.59
	*Protective factors*												
	Non-smoking pregnancy	**0.98**	**[0.96,**	**0.99]**	**0.002**	0.98	[0.94,	1.02]	0.41	0.01	[0.00,	0.02]	0.10
	No alcohol used in pregnancy	**1.04**	**[1.00,**	**1.08]**	**0.047**	**1.15**	**[1.03,**	**1.28]**	**0.01**	-0.02	[-0.05,	0.00]	0.09
	Higher social-emotional wellbeing	1.00	[0.98,	1.01]	0.61	1.01	[0.98,	1.05]	0.48	0.00	[-0.01,	0.01]	0.78
	Cultural-based resilience	**0.79**	**[0.66,**	**0.95]**	**0.01**	0.92	[0.55,	1.54]	0.74	**0.12**	**[0.01,**	**0.24]**	**0.03**
	Fewer stressful life events	**1.38**	**[1.08,**	**1.76]**	**0.01**	0.64	[0.34,	1.20]	0.16	-0.05	[-0.19,	0.10]	0.55
	Maternal age	**0.83**	**[0.69,**	**0.99]**	**0.04**	1.73	[0.86,	3.47]	0.13	**0.51**	**[0.38,**	**0.64]**	**<0.001**
	Maternal education Year 12 or above	1.08	[0.91,	1.29]	0.36	1.08	[0.65,	1.81]	0.76	**-0.13**	**[-0.24,**	**-0.01]**	**0.03**
	Accessible geographical location	1.00	[0.99,	1.00]	0.28	**0.98**	**[0.96,**	**0.99]**	**0.01**	**-0.02**	**[-0.02,**	**-0.01]**	**<0.001**
	Mother Aboriginal or Torres Strait Islander	0.90	[0.77,	1.06]	0.22	**0.58**	**[0.35,**	**0.97]**	**0.04**	-0.09	[-0.20,	0.02]	0.11
	Mother employed	1.34	[0.61,	2.96]	0.46	0.40	[0.12,	1.32]	0.13	-0.29	[-0.74,	0.17]	0.22

*Notes*: BMI = body mass index. Data in tables are from data including multiple imputation and are odds ratios (OR) with 95% confidence intervals [CI] from logistic regression [global physical health] reflecting the relative odds of being in a less healthy group; logistic regression [disability]; or regression coefficients (coef) with 95% CI from multiple linear regression [BMI]. Bold-face represents statistically significant results (p<0.05).

Very good or excellent global child health were experienced more frequently in context of a range of protective pregnancy and environmental factors, including mothers not smoking during pregnancy, higher levels of maternal-reported cultural-based resilience, and older maternal age. Cultural-based resilience and older maternal age were similarly protective against low BMI. Childhood disability was less frequently reported by mothers in less geographically remote regions. While the results showed that mothers abstaining from alcohol and experiencing lower life stress were less likely to report that their child had very good or excellent health, and mothers abstaining from alcohol were more likely to report that their child had a disability, it is more likely that these findings are reflecting higher health literacy and early recognition of children’s difficulties for these mothers than a negative effect.

## Discussion

Our findings suggest there is a continued long-term health burden associated with perinatal risk in this recent cohort of Aboriginal and Torres Strait Islander children. The most evident concern was that children born with moderate-to-high perinatal risk were much more underweight than full-term children, on average by almost half a standard deviation, and this was not explained by the child or environmental factors considered. Effects for mild perinatal risk were smaller, but still showed an association with lower body weight not explained by child or environmental factors. Perinatal risk was associated with poorer global health although associations were no longer evident once child and environmental factors were considered. Our findings suggest that mothers not smoking during pregnancy and higher levels of maternal-reported cultural-based resilience are protective factors for children’s health outcomes.

Persistent underweight represents a risk to healthy development in general populations, and is associated with poorer global health and special health-care needs in younger children.[21] Our findings are consistent with the follow-up data from the Aboriginal Birth Cohort Study, investigating BMI outcomes at age 8–14 years in children born small for gestational age in 1987–1990. This study followed up 279 of the original birth cohort of 686 Aboriginal and Torres Strait Islander children, and found that all children had a negative mean z-score for height and weight; but children born low birth weight remained more underweight than peers in childhood. They also found that child size rather than birthweight predicted childhood insulin and glucose metabolism.[22] This cohort was followed again at age 18, where children born small continued to be smaller and lighter.[23] As these authors argue, it will be important for future research to track health outcomes consistently from early childhood through to later adulthood to determine whether the effects of perinatal risk change over the life course, and how childhood underweight translates to adult health (such as overweight and obesity) in later life. Low maternal BMI has been shown to be a predictor of preterm birth in women attending Townsville Aboriginal and Islander Health Services,[24] thus there may be danger of cyclical risk over multiple generations. One potential avenue for further investigation may be to model individual BMI trajectories with repeated measures data across childhood.

Our findings for child physical health and disability are less easily interpreted than those related to BMI. Three-quarters of mothers in the overall sample rated their child as having very good or excellent global health. Although there were no significant differences between perinatal risk groups for global physical health in the final adjusted models, a gradient was evident whereby children with perinatal risk, and particularly those with moderate-to-high risk, were more likely to have lower global ratings indicating poorer health compared to children born healthy term. Similarly, children with moderate-to-high perinatal risk were more likely to have a disability, although large variability in the data (related to low prevalence of disability conditions) meant that this difference was not statistically significant. Both outcomes were assessed using single-item mother-reported measures rather than symptom-based measures or screening instruments, which may have reduced recognition in the sample. Under-recognition and diagnosis of some conditions may relate to how health services vary between communities, and/or problems with the appropriateness of these health questions in relation to Aboriginal and Torres Strait Islander language and culture.[25] Further investigation is required.

The examination of potential environment protective factors in the current study identified a number of pathways by which the risks associated with being born preterm, low birth weight or small for gestational age may be mitigated. Our findings supported cultural-based resilience being protective against poor global physical health outcomes and low BMI. Not smoking during pregnancy was also associated with better global physical health. Child health outcomes may also be better for older mothers and those living in less remote geographical areas.

Although there has been limited research investigating long-term child health outcomes following preterm, small for gestational age or low birth weight in other Indigenous populations around the world, there are strong similarities in the health outcomes for Australian Aboriginal and Torres Strait Islander populations and in other Indigenous populations around the world. For example, rates of preterm birth are 40–70% higher in First Nation and Inuit children in Canada; in American Indian, Native Hawaiian and Alaskan Native Peoples in the United States, and in Maori People in New Zealand, compared to their non-Indigenous counterparts in the same countries.[26] These populations also carry a higher burden of disease and poor health outcomes compared to non-Indigenous populations.[26] A key strength of the current study was the large national sample of Aboriginal and Torres Strait Islander children followed prospectively. However, generalisability may be limited as our analyses required families to have complete perinatal data. The context for current Aboriginal and Torres Strait Islander families may have also changed since recruitment in 2008 due to numerous factors, including those related to policy changes and government initiatives. For example, smoking rates in Aboriginal people have declined since 2001 but remains high [27] and differs greatly by region [28]. Although, these kind of positive health improvements over the decade are unlikely to influence the associations between variables, they may have resulted in fewer families and children affected.

The rate of missing data for child BMI was high at 26%. Although we used multiple imputation, it is possible that the imputation models based on the available data did not fully account for missing data and our findings may not be representative. Further, it was not possible to differentiate outcomes for different communities of Aboriginal and Torres Strait Islander children, or for children living in urban versus rural or remote areas, which we would recommend as an important step for the future.[29] In considering environmental factors as well as perinatal risk outcomes we were able to find evidence supporting not smoking through pregnancy and cultural-based resilience being protective factors for children’s health. We recognise that differences in early recognition and reporting of children’s difficulties may have masked expected associations between the use/or abstinence of alcohol and children’s health outcomes.[30–32]

A key limitation of accessing an existing national dataset applies to our study, where the authors did not have input into the design or selection of measures in LSIC. The LSIC study relied on retrospective collection of birth data during infancy or childhood. All findings require further examination with prospective data, and future research should also validate the use of the physical health outcome measures with an Aboriginal and Torres Strait Islander population with more in-depth measures and/or via direct assessment with a physician. We acknowledge the potential limitation represented in our data by using Australian norms for categorisation of our birthweight data, while the child BMI data uses international norms taken from a World Health Organisation study. It’s not clear what influence this may have had on our findings, but future studies should consider using one system consistently. Our study also only focussed on a specific subset of perinatal risks related to children being born small and or early. There is also research evidence showing increased poor health outcomes including obesity associated with high birth weight[33], which will be an important area for to investigate for health promotion within the Australian Aboriginal and Torres Strait Islander population.

In conclusion, our study provides an assessment of perinatal risk in a recent cohort of Aboriginal and Torres Strait Islander children. The rates of perinatal risk in this sample were very high, with almost one third of the sample born preterm, low birthweight and/or small for gestational age. These rates are comparable to those from cohorts born 20–30 years earlier,[34] and demonstrate that these risks have persisted despite increased attention on Indigenous health over the past decades. Our findings show that moderate-to-high perinatal risk is associated with childhood underweight and with poorer global physical health, although the latter finding requires further examination. These results are concerning and indicate potential health disparities for Aboriginal and Torres Strait Islander children born with perinatal risk. The best opportunity to reduce long-term health disparities may be a comprehensive approach focussed both on addressing social adversity and improving health service access and provision, particularly for women at high risk or in remote areas.[35–37] Our study identified two modifiable protective factors associated with better child health outcomes that suggest future efforts should focus on improving pre-pregnancy care, including supporting women’s smoking cessation and increasing cultural-based resilience related to socio-emotional wellbeing and connection to community.[38] In particular, strategies focussed on increasing cultural-based resilience may help to ameliorate the association between perinatal risk and poor child health outcomes. Our findings are consistent with the National Aboriginal and Torres Strait Islander Health Plan for 2013–2023, which emphasizes the importance of strengthening communities, reinforcing positive behaviours, and improving living conditions to support better health.[9]
